# NMDA receptor-dependent regulation of miRNA expression and association with Argonaute during LTP *in vivo*

**DOI:** 10.3389/fncel.2013.00285

**Published:** 2014-01-13

**Authors:** Balagopal Pai, Taweeporn Siripornmongcolchai, Birgitte Berentsen, Ashraf Pakzad, Christel Vieuille, Ståle Pallesen, Maciej Pajak, T. Ian Simpson, J. Douglas Armstrong, Karin Wibrand, Clive R. Bramham

**Affiliations:** ^1^Department of Biomedicine and K.G. Jebsen Centre for Research on Neuropsychiatric Disorders, University of BergenBergen, Norway; ^2^Department of Psychosocial Science, University of BergenBergen, Norway; ^3^Institute for Adaptive and Neural Computation, School of Informatics, University of EdinburghEdinburgh, UK; ^4^Biomathematics and Statistics ScotlandJCMB, Edinburgh, UK

**Keywords:** synaptic plasticity, microRNA, RNA-induced silencing complex, Argonaute, microRNA target prediction, gene expression, protein synthesis, hippocampus

## Abstract

microRNAs (miRNAs) are major regulators of protein synthesis in the brain. A major goal is to identify changes in miRNA expression underlying protein synthesis-dependent forms of synaptic plasticity such as long-term potentiation (LTP). Previous analyses focused on changes in miRNA levels in total lysate samples. Here, we asked whether changes in total miRNA accurately reflect changes in the amount of miRNA bound to Argonaute protein within the miRNA-induced silencing complex (miRISC). Ago2 immunoprecipitation was used to isolate RISC-associated miRNAs following high-frequency stimulation (HFS)-induced LTP in the dentate gyrus of anesthetized rats. Using locked-nucleic acid-based PCR cards for high-throughput screening and independent validation by quantitative TaqMan RT-PCR, we identified differential regulation of Ago2-associated and total miRNA expression. The ratio of Ago2/total miRNA expression was regulated bidirectionally in a miRNA-specific manner and was largely dependent on N-methyl-D-aspartate receptor (NMDA) activation during LTP induction. The present results identify miRNA association with Ago2 as a potential control point in activity-dependent synaptic plasticity in the adult brain. Finally, novel computational analysis for targets of the Ago2-associated miRNAs identifies 21 pathways that are enriched and differentially targeted by the miRNAs including axon guidance, mTOR, MAPK, Ras, and LTP.

## Introduction

Stable forms of activity-dependent synaptic plasticity require coordinated gene transcription and bursts of protein synthesis and degradation. Rapid regulation of local protein synthesis in dendrites is considered important for synaptic homeostasis and plasticity (Bramham and Wells, 2007; Martin and Ephrussi, 2009). In recent years miRNAs have emerged as key modulators of neuronal protein synthesis. miRNAs are short non-coding RNAs (~22 nucleotides) that bind to partially complementary sites on the 3′ UTR of target mRNAs where they act to inhibit protein synthesis (Filipowicz et al., 2008; Djuranovic et al., 2011; Huntzinger and Izaurralde, 2011). Many new brain-specific miRNAs have appeared with vertebrate and primate evolution and roles for specific miRNAs in neurogenesis, dendritic spine morphogenesis, synaptic regulation, plasticity, and memory storage have been demonstrated (Vo et al., 2005; Krichevsky et al., 2006; Rajasethupathy et al., 2009; Siegel et al., 2009; Mellios et al., 2011; Tognini et al., 2011). The target diversity, specificity, and activity-dependent regulation make miRNAs attractive as modulators of local protein synthesis and synaptic plasticity.

In the canonical biogenesis pathway, miRNAs are transcribed as long primary transcripts and processed in the nucleus by the RNase III enzyme Drosha to generate a stem-loop structured precursor. The precursor miRNA is then exported to the cytoplasm where a second RNase III enzyme, Dicer, generates a mature double-stranded miRNA intermediate. One of these strands, the guide strand, is recognized and bound by the protein Argonaute (Ago), which further functions in recruitment of the multi-protein, miRNA-induced silencing complex (miRISC) (Bartel, 2004). The passenger strand of the miRNA duplex is normally destroyed. Once assembled on target bound miRNA, the miRISC inhibits protein synthesis by repressing translation, promoting mRNA decay, or some combination of the two processes (Filipowicz et al., 2008; Huntzinger and Izaurralde, 2011; Béthune et al., 2012; Djuranovic et al., 2012).

MicroRNA activity is modulated through changes in miRNA biogenesis, miRNA turnover, and regulation of RISC effector proteins (Lugli et al., 2005; Ashraf et al., 2006; Kosik, 2006; Banerjee et al., 2009; Krol et al., 2010a,b; Wibrand et al., 2010) A previous study on miRNA expression in dentate gyrus long-term potentiation (LTP) provided evidence for rapid, activity-dependent decay of several mature miRNAs in addition to upregulation of miRNA by transcription (Wibrand et al., 2010). In that study and previous work on chemically induced LTP (Park and Tang, 2009), miRNA levels were measured in whole tissue lysate. The general assumption has been that guide-stranded mature miRNA is predominantly, if not exclusively, bound to Argonaute. However, mounting evidence suggest that miRNA binding to Argonaute is both reversible and regulated (Meister, 2013).

Using Ago2 immunoprecipitation, we demonstrate rapid, differential regulation of Ago2-associated and total miRNAs following LTP induction in the dentate gyrus *in vivo*. The ratio of Ago2/total miRNA expression was regulated bidirectionally in a miRNA-specific manner and was coupled to N-methyl-D-aspartate (NMDA) receptor-dependent LTP induction. Hence, these findings identify regulation of miRNA:Ago2 interactions as a potential control point in long-term synaptic plasticity of the adult dentate gyrus.

Finally we use an integrated miRNA target prediction approach to inform pathway enrichment analyses in which we find that many of the major pathways traditionally associated with the regulation of synaptic plasticity are targeted, including the mTOR, MAPK, and Ras pathways. Surprisingly, the axon guidance pathway is the most highly enriched, but includes many genes already known to play a role in synaptic plasticity.

## Materials and methods

### *In vivo* electrophysiology

Experiments were carried out under ethical standards approved by the Norwegian Committee for Experiments on Animals. *In vivo* electrophysiological experiments were carried out on 30 adult male rats of the Sprague–Dawley outbred strain, weighing 250–350 g. The electrophysiological procedures have been detailed elsewhere (Messaoudi et al., 2002; Panja et al., 2009). Rats were anesthetized with urethane and electrodes were stereotactically placed for selective stimulation of the medial perforant pathway and recording of evoked field potentials in the hilar region of the dentate gyrus. A concentric bipolar stimulating electrode (input impedance ~40 MΩ; tip separation 500 μm; SNEX 100; Rhodes Medical Instruments, Woodland Hills, CA) was lowered into the dorsomedial aspect of the angular bundle for stimulation of the medial perforant path.

The recording electrode was slowly lowered into the dorsal hippocampus until a positive-going field EPSP (fEPSP) of maximum slope was obtained in the dentate hilus (7.9 mm posterior to bregma and 4.2 mm lateral from midline for stimulation; 3.9 mm posterior and 2.3 mm lateral for recording). Biphasic rectangular test pulses of 150 μs duration were applied every 30 s throughout the experiment (0.033 Hz) except during the period of high-frequency stimulation (HFS). Responses were allowed to stabilize, and 20 min of baseline recording was obtained. HFS consisted of 400-Hz, 8-pulse stimulus trains repeated 4 times with 10 s between each train. HFS was applied three times with 5 min between each session. The total number of pulses was 128. After HFS, evoked responses were collected for periods of 30 and 120 min. Signals from the dentate hilus were amplified, filtered (1 Hz and 10 Hz), and digitized (25 Hz). Acquisition and analysis of field potentials were accomplished using Data Wave Technologies Work Bench Software (Longmont, CO). The maximum slope of the fEPSP was measured and the averages of four consecutive responses were obtained.

### Intrahippocampal infusion

The recording electrode with an attached guide cannula was lowered into the dentate gyrus as described for the recording electrode above. An inner infusion cannula (31 gauge) was then inserted so it protruded 300 μm below the end of the guide. The tip of the infusion cannula was located in the deep stratum lacunosum-moleculare of field CA1, 700 μm above the hilar recording site and 300–400 μm above the medial perforant synapse (Messaoudi et al., 2007). The inner infusion cannula was connected via a polyethylene (PE50) tube to a 3 μl Hamilton syringe (Reno, NV) and infusion pump. 0.3 μl of 2-Amino-5-phosphonopentanoic acid (AP5, 50 mM prepared in 1× PBS; Tocris) was infused over 12 min at a rate of 0.085 μl/min, and test pulse stimulation was continued for a further 18 min prior to HFS.

### Dentate gyrus dissection and sample preparation

At the end of the electrophysiological recordings rats were decapitated, the brain was extracted and rinsed with ice-cold saline and both hippocampi were removed within less than 3 min. The dentate gyri were then rapidly dissected on ice and immediately frozen on dry ice.

### Argonaute 2 immunoprecipitation

Immunoprecipitation was performed according to Choe et al. (2010) with minor modifications. Forty microliters of protein G-sepharose (17061801, GE Health Care Bioscience AB) was incubated with 3 μg of mouse monoclonal Ago2 antibody (anti-EIF2C2) (H00027161-M01, Abnova) at room temperature in a rotary shaker (25 rpm) for 1.5 h. Immunoprecipitation using purified mouse IgG (558509, BD Pharmingen) served as a control for detection of non-specific protein binding. The sepharose beads were blocked with 2% yeast tRNA (R4018, Sigma-Aldrich) and 1% BSA (9048-46-8, Sigma-Aldrich) in 1× PBS at room temperature in a rotary shaker (25 rpm) for 30 min. Dentate gyri were homogenized (Dounce homogenizer) in ice cold lysis buffer containing 25 mM Tris (Ambion Life Sciences, Carlsbad, CA, USA), 150 mM NaCl, 2 mM MgCl2, 0.5% NP-40 (Ambion Life Sciences, Carlsbad, CA, USA), 0.5 mM DTT (Ambion Life Sciences, Carlsbad, CA, USA), 0.4 unit RNAse inhibitor (Ambion Life Sciences, Carlsbad, CA, USA), and protease inhibitor cocktail (Roche Diagnostics GmbH) 1 tablet/10 ml. The homogenate was centrifuged at 4°C (10,000 rpm, 15 min) and subjected to pre-clearing with 40 μl protein-G sepharose at 4°C for 30 min, and centrifuged at 3000 rpm for 3 min. The protein concentration of the pre-cleared supernatant was determined by the BCA Protein Assay (BCA Protein Assay kit 23227, Thermo Scientific). A small aliquot of the supernatant was separately stored at 4°C for analysis of total lysate (input). The antibody bound beads were incubated with 750 μg of pre-cleared protein at 4°C in a rotary shaker (25 rpm) for 2.5 h. Non-specifically bound proteins were removed from the sepharose beads by giving four washes: first wash using lysis buffer, second wash with lysis buffer containing 900 mM NaCl instead of 150 mM, a third wash in the standard lysis buffer, and a final wash in lysis buffer containing 0.05% NP-40.

### RNA isolation

The Ago2 immunoprecipitate (referred to as Ago2 IP or Ago2 pellet) and the input samples were treated with 250 μl DNAse solution containing 25 μl of 10× DNAse1 buffer, 1 unit DNAse1 (Ambion Life Sciences, Carlsbad, CA, USA) and 50 unit RNAse inhibitor (Ambion Life Sciences, Carlsbad, CA, USA) at 37°C for 20 min for genomic DNA removal. Both the input (containing total RNA including all miRNAs) and the Ago2 IP (containing all RNAs in Ago2 complex including Ago2-associated miRNAs) were then treated with 500 μl of Trizol (Ambion Life Sciences, Carlsbad, CA, USA) for 5 min and 200 μl of Chloroform (Sigma-Aldrich, St. Louis, MO, USA), and subjected to centrifugation at 4°C, 14,000 rpm for 15 min. The water phase containing RNA was collected. RNA was precipitated in a solution containing 500 μl isopropanol, 5 μg glycogen (Ambion Life Sciences, Carlsbad, CA, USA), and 10% v/v ammonium acetate (Ambion Life Sciences, Carlsbad, CA, USA) at –20°C for at least 16 h. The preparation was then centrifuged at 4°C, 14,000 rpm for 45–60 min. The pellet was washed once in 100% ethanol, and once in 80% ethanol prior to centrifugation at 4°C, 14,000 rpm for 20 min. The RNA pellet was air-dried for 15 min, and dissolved in 25 μl of nuclease-free water. The quantity and quality of the RNA were determined by absorbance measurement at 260/280 nm. The quality of the total RNA was assessed by PCR-Quality Control (PCR-QC) using TaqMan® Gene expression assays (Applied Biosystems, Life Sciences, Carlsbad, CA, USA) for amplification of miRNAs which are known to express in the brain: miR-347 (assay 1334) and miR-151 (assay 1330). Argonaute immunoprecipitation was validated by western blot analysis of Ago2 protein in cortex, cornu ammonis, dentate gyrus, and HEK293 cells transfected with GFP-Ago2 vector.

### High-throughput PCR card analysis of microRNA expression

High-throughput PCR analysis was performed using the miRCURY LNA universal RT miRNA Ready-to-Use PCR Mouse and Rat panel 1, V2.R (PCR card 203706, Exiqon). Twenty nanograms total RNA was reverse-transcribed to cDNA according to the universal cDNA synthesis kit instructions (203300, Exiqon). The qPCR was performed according to the SYBR Green Master Mix Universal RT kit instructions (203400, Exiqon). The PCR reaction was performed on a Roche Light Cycler® 480 II (Roche Applied Science). Briefly, a poly-A tail is added to the mature miRNA template. The cDNA template is then amplified using miR-specific LNA™ enhanced forward and reverse primers that are pre-aliquoted in the PCR card. The reverse primers detect the poly-T tail and the 3′ part of the mature microRNA sequence, ensuring specific amplification of the mature microRNA.

Of 384 wells in the PCR card, 373 wells contain different primer sets for 373 miRNAs of known sequences present in mirBase version 16. In addition, 11 wells are used for the following controls: an empty well (blank), three interplate-calibrators, a primer pair specific to synthetic oligo-RNA template in the cDNA synthesis master mix (for quality control of cDNA synthesis), and wells for the six reference genes miR-423-3p, miR-103, miR-191, u6 (small nucleolar RNA), RNU-5G (small nucleolar RNA), and rnu-1A1 (small nucleolar RNA).

The data analysis was performed according to the manufacturer's instructions (Exiqon GenEx qPCR analysis software). For each of three rats, the Ct (threshold cycle) values from the input and Ago2 IP samples collected at 30 and 120 min post-HFS were normalized using global normalization, an appropriate approach for large scale analysis (>100 assays) in which most miRNAs are not regulated. The Ct value of each miRNA was normalized to the mean Ct of all the miRNAs represented on the PCR card. Differences in miRNA expression between the treated and contralateral control dentate gyrus were compared by a Student's *t*-test with Dunn–Bonferroni correction (GeneEx software). One-Way ANOVA and *post hoc* Fisher's LSD was additionally applied to the data analysis in order to allow detection of possible false-negative results from Dunn–Bonferroni correction.

### TaqMan real-time RT-PCR analysis of microRNA

Ten nanograms total RNA was reverse-transcribed to cDNA according to the TaqMan® miRNA reverse transcription Kit instructions (4366597, Applied Biosystems) provided along with the TaqMan® RT primer specific to miRNA of interest. TaqMan® RT primers (Applied Biosystems) RT00-2602 (miR-384-5p), RT00-0413 (miR-29b), RT00-0522 (miR-219), RT00-2017 (miR-592), RT00-0580 (miR-20a), RT00-0382 (let-7f), RT00-2230 (miR-330-5p), RT00-0548 (miR-338), RT00-0526 (miR-223), RT00-2551 (miR-212), RT00-0480 (miR-181a), RT000426 (miR-34a), RT000395 (miR-19a), RT001061 (miR-326), and RT00-0492 (miR-193a) were used.

To identify the most stable gene for normalization, multifactor ANOVA for multiple comparisons was used to analyze the Ct (threshold cycle) values of 373 miRNAs in Ago IP at 30 and 120 min and in input at 30 and 120 min (from the PCR cards). Two genes (miR-345-3p and miR-214) with most stable expression across samples (with highest *P*-value and lowest SD in Multi-Factor ANOVA analysis) were tested by qPCR in Ago2 IP (*n* = 6) and input samples (*n* = 6) in both treated and control dentate gyrus obtained at 30 min post-HFS. Rno-miR-345-3p was found to be the most stably expressed with the lowest range of Ct values among all samples, and therefore was used as an optimal normalizer in qPCR validation of miRNA expression.

cDNA pre-amplification was performed to allow accurate and reliable qPCR analyses of low abundance miRNAs. Fourteen cycles of exponential phase cDNA pre-amplification was performed according to the TaqMan® PreAmp Master Mix instructions (4391128, Applied Biosystems). The pre-amplified cDNA was diluted 10 times and subjected to qPCR. The qPCR was performed on a Roche Light Cycler®480 II (Roche Applied Science) using diluted pre-amplified cDNA from the individual dentate gyrus input and Ago2 IP samples. After reverse transcription and pre-amplification, qPCR was analyzed in a 25 μ l reaction volume using 2× TaqMan® Universal PCR Master Mix II with no uracil-N-glycosylase (Applied Biosystems). PCR quantification was performed in triplicate using the relative standard curve method to determine gene expression levels. TaqMan®Gene expression assays (Applied Biosystems) were used corresponding to the TaqMan® RT primers used in cDNA synthesis.

### Quantitative PCR analysis of *Arc* mRNA

For analysis of *Arc* mRNA, 2 μg total RNA was reverse-transcribed to cDNA according to the MMLV reverse transcriptase kit instructions (2043, Ambion). qPCR was performed on a Roche Light Cycler® 480 II (Roche Applied Science) using cDNA corresponding to 10 ng total RNA of individual homogenized dentate gyrus. The qPCR was analyzed in 25 μ l reaction using 2×TaqMan® PCR mix (Applied Biosystems). The qPCR quantification was performed in triplicate using relative standard curve method to determine gene expression levels. Ubiquitin-B was used as a housekeeping gene to normalize and determine the expression level for *Arc*. TaqMan®Gene expression assays (Applied Biosystems): Rn00571208_g1 (*Arc*) and Rn03062801_g1 (ubiquitin-B) were used.

### Statistical analysis for qPCR validation

Statistical analyses were performed using SPSS (version 16). Two miRNAs (miR-181a and miR-193a) were undetectable in the control dentate gyrus because their Ct values were greater than 40, and were therefore excluded from the analyses. One-Way ANOVA was used to analyze relative expression differences between the treated and contralateral control dentate gyrus in both Ago2 IP and input for all miRNAs. The expression levels between different miRNAs, as well as the differences between treatment groups were analyzed using a One-Way ANOVA and Fisher's LSD *post hoc* test. Fold changes in treated dentate gyrus relative to the contralateral control dentate gyrus were calculated for Ago2 IP and input samples from the individual rats and averaged.

### microRNA target prediction

To identify the putative target genes of each miRNA we first queried four of the most widely used target prediction sources; DIANA (Maragkakis et al., 2011), miRanda (Griffiths-Jones et al., 2008), TargetScan (Friedman et al., 2009), and PicTar (Lall et al., 2006). Both DIANA and PicTar report miRNA targets in *Mus musculus* which we projected into *Rattus norvegicus* using only direct orthologs extracted from the Ensembl Compara database (Vilella et al., 2009).

We next quantified the agreement between predicted target lists using Rank Product (RP) analysis (Breitling et al., 2004; Eisinga et al., 2013). Briefly, each gene was ordered by quality score and the geometric mean of the gene rank calculated across prediction sources. Missing ranks were imputed for target genes missing only one rank value, genes missing more source values were discarded. To assess the robustness of the computed ranks we performed a bootstrap analysis with 1000 permutations of rank order using the Bioconductor RankProd package (Hong et al., 2006). In order to minimize the elimination of true positive targets, genes with RP (*p* > 0.5) were used in subsequent pathway analysis.

### Pathway analysis

Integrated miRNA target gene lists from the RP step were used as input to pathway enrichment analyses by hypergeometric testing using the Bioconductor KEGGprofile package (Zhao, 2012). Gene to KEGG pathway mappings were retrieved for every pathway in the KEGG database (Kanehisa et al., 2011) and used to identify pathways that were enriched in predicted miRNA target genes (*p* ≤ 0.05).

## Results

### Validation of Argonaute-2 immunoprecipitation

**Figure 1:**
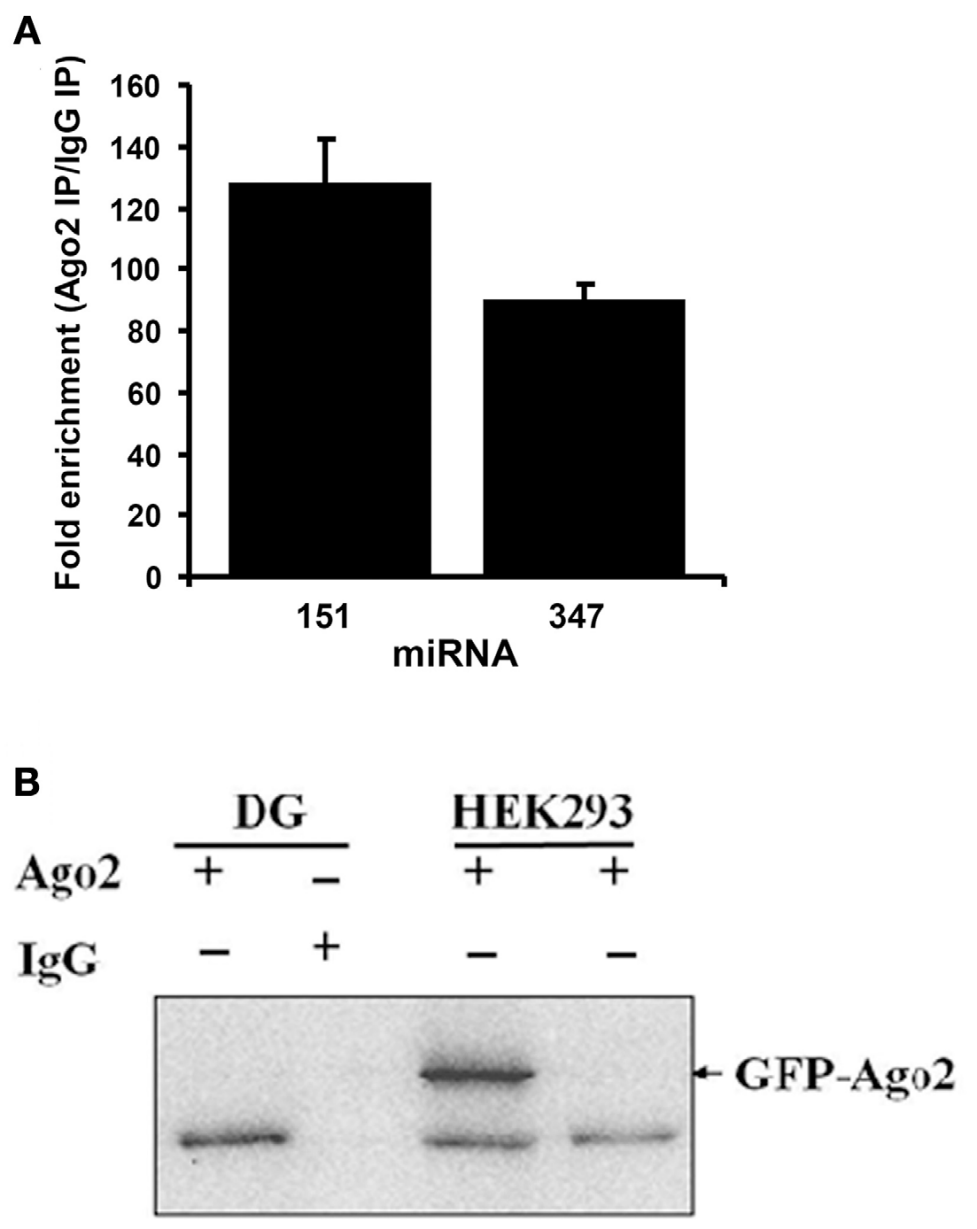
**Validation of Argonaute 2 immunoprecipitation. (A)** Specific Ago2-immunoprecipitation of brain-enriched miRNAs. TaqMan qPCR for brain-enriched miR-347 and miR-151 was performed in Ago2 immunoprecipitated (IP) or non-immune mouse IgG immunoprecipitated samples from dentate gyrus. Values are means (+s.e.m.; *n* = 3) expressed as fold enrichment in the Ago2 IP relative to IgG IP. **(B)** Ago2 immunoblots following immunoprecipitation of dentate gyrus (DG) samples with Ago2 antibody or non-immune mouse IgG. A band at ~97 kDa corresponding to Ago2 was detected in Ago2, but not IgG, IP. As a positive control, HEK293 cells were transfected with GFP-Ago2. Endogenous Ago2 protein (lower band) and GFP-Ago2 fusion protein (arrow; band at ~127 kDa) were detected in transfected cells.

Dentate gyrus lysates were immunoprecipitated with an Ago2-specific antibody or control, non-immune IgG, and expression of the brain-specific miRNAs, miR-151, and miR-347, was examined by qPCR. miR-151 and miR-347 levels were enriched more than 100-fold in the Ago2 IP (Figure 1A). Furthermore, western blot analysis detected Ago2 protein at 97 kDa in the Ago2 pellet from naïve rat dentate gyrus, but not in the non-immune IgG pellet (Figure 1B). As a positive control, ectopically expressed Ago2-GFP was detected in HEK293 cells (Figure 1B). The results confirm immunoprecipitation of Ago2 and Ago2-associated miRNAs.

### High-throughput miRNA expression profiling

**Figure 2:**
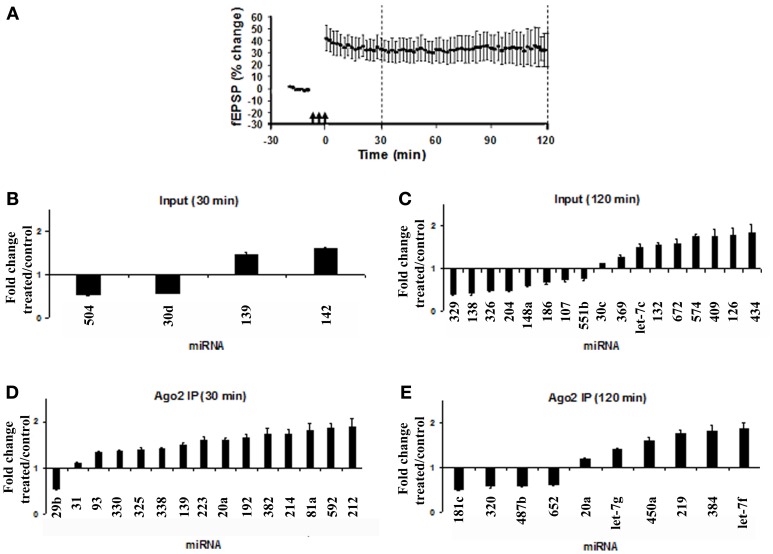
**Modulation of miRNA expression in total lysates and Ago2 immunoprecipitates following LTP induction. (A)** Time course plot showing changes in the medial perforant path-evoked fEPSP slope expressed as a percentage of baseline. Values are mean ± s.e.m. (*n* = 3). Dentate gyrus tissue was obtained at 30 or 120 min post-HFS. **(B–E)** miRNA PCR cards from Exiqon were used to screen 373 miRNAs in total lysates (input) and Ago2 immunoprecipitates following high-frequency stimulation (HFS). Data for input and Ago2 IP samples for each time point were normalized to the respective global mean of all miRNAs expressed on the card. Values are mean (+s.e.m.) changes in HFS-treated dentate gyrus relative to the contralateral control (*n* = 3). Values are significantly different from control (Student's *t*-test with Dunn–Bonferroni correction, *p* < 0.05). **(B)** Fold change in miRNA expression in dentate gyrus input at 30 min post-HFS. **(C)** Fold change in miRNA expression in dentate gyrus input at 120 min post-HFS. **(D)** Fold change in miRNA expression in dentate gyrus Ago2 immunoprecipitate at 30 min post-HFS. **(E)** Fold change in miRNA expression in dentate gyrus Ago2 immunoprecipitates at 120 min post-HFS.

Brief bursts of HFS applied to the medial perforant path of anesthetized rats induced stable LTP of the fEPSP slope (Figure 2A). The dentate gyrus was rapidly micro-dissected at 30 or 120 min post-HFS. miRNA expression in the HFS-treated dentate gyrus was compared with the contralateral, unstimulated dentate gyrus which served as an internal control. High-throughput expression profiling of Ago2 IP and input samples was performed using pre-aliquoted, locked-nucleic acid based miRNA PCR primer sets in 384-well PCR plates. As there is no known reference gene stably expressed in both the Ago2 pellet and input, a global normalization was performed in which the Ct value of each miRNA was normalized to the mean Ct of all the miRNAs represented on the PCR card. Statistical differences in miRNA expression between the treated and control dentate gyrus were assessed by a Student's *t*-test with Dunn–Bonferroni correction.

In dentate gyrus lysates (inputs), at 30 min post-HFS, only 4 of 376 miRNAs represented on the panel exhibited significantly altered expression. Fold-changes ranged between a decrease of 0.49-fold (miR-504) and an increase of 1.55-fold (miR-142) relative to the contralateral control dentate gyrus (Figure 2B). At 120 min post-HFS, 17 miRNAs were significantly regulated, ranging between a maximum decrease of 0.65 fold (miR-329) and maximum increase of 1.79 fold (miR-434) (Figure 2C). In agreement with previous results (Wibrand et al., 2012), miR-132 was significantly upregulated at 120 min post-HFS in dentate gyrus lysates (Figure 2C). The high-throughput analysis of input samples indicate predominantly delayed (2 h), bidirectional changes in miRNA expression following HFS.

In Ago2 IP samples, 14 miRNAs were upregulated at 30 min post-HFS, and only miR-29b was downregulated (Figure 2D). At 120 min, 10 miRNAs were regulated (Figure 2E). None of the miRNAs exhibiting altered expression in the Ago2 pellet at 30 or 120 min post-HFS were also regulated in the input samples. Thus, the PCR card analysis revealed rapid, differential regulation of miRNAs in the Ago2-immunoprecipitated fraction relative to total miRNA.

### LTP-specific, differential regulation of Ago2-associated and total miRNA expression: qPCR validation

**Figure 3:**
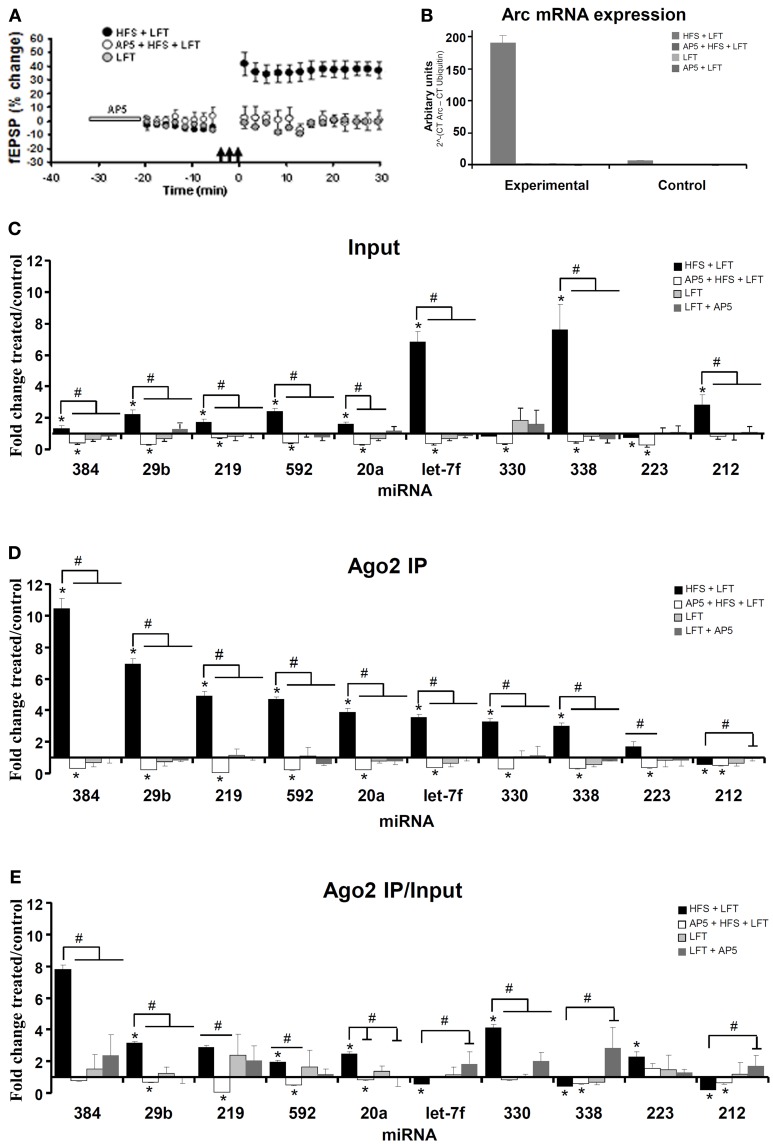
**Differential expression of Ago2-associated and total miRNAs linked to NMDA receptor-dependent LTP induction. (A)** Time course plot showing changes in the medial perforant path-evoked fEPSP slope expressed as a percentage of baseline. Values are mean (±s.e.m.). HFS + LFT, *n* = 6; AP5 + HFS + LFT, *n* = 4; LFT, *n* = 3. Dentate gyrus tissue was obtained at 30 min. **(B)** Quantitative PCR was used to validate the expression of *Arc* mRNA. Changes in *Arc* mRNA levels in the treated and contralateral control dentate gyrus lysate samples were analyzed. The PCR data was normalized to the expression of *Ubiquitin-B* using the ΔCt method. Values are mean (+s.e.m.). HFS + LFT, *n* = 6; AP5 + HFS + LFT, *n* = 4; LFT, *n* = 3: AP5 + LFT, *n* = 5. **(C–E)** Quantitative TaqMan PCR was used for independent analysis of 10 selected miRNAs from the PCR card screen. The same set of miRNAs were analyzed in the input sample **(C)** and Ago2-immunoprecipitate **(D)**. qPCR data was normalized to expression of miR-345-3p. **(C)** Fold change in miRNA expression in dentate gyrus input samples at 30 min. Bar graph shows mean fold change (+s.e.m.) in treated dentate gyrus relative to control, contralateral dentate gyrus. Significant differences between the HFS group and other treatment groups are indicated (#; *p* < 0.05). Significant difference between the ispilateral (treated) and contralateral (control) dentate gyrus are indicated (^*^). HFS + LFT, *n* = 6; AP5 + HFS + LFT, *n* = 4; LFT alone, *n* = 5; AP5 + LFT, *n* = 5. **(D)** Fold change in miRNA expression in dentate gyrus Ago2 immunoprecipitates at 30 min post-HFS. Significant differences between the HFS group and other treatment groups are indicated (#; *p* < 0.05). Significant difference between the ispilateral (treated) and contralateral (control) dentate gyrus are indicated (^*^). **(E)** Relative fold change in miRNA expression in dentate gyrus Ago2 immunoprecipitates compared to dentate gyrus lysates (Ago2/input expression ratios) at 30 min post-HFS. Bar graph shows relative fold change in treated dentate gyrus relative to control, contralateral dentate gyrus. Significant differences between the HFS group and other treatment groups are indicated (#; *p* < 0.05). Significant difference between the ispilateral (treated) and contralateral (control) dentate gyrus are indicated (^*^).

TaqMan-based qPCR analysis of independent samples was used to validate changes in Ago2-immunoprecipitated miRNAs at 30 min post-HFS. In the qPCR validation, four experimental groups were used in order to identify miRNA regulation specific to LTP induction. The treatment groups were: (1) HFS + LFT; *n* = 6, (2) block of LTP induction by local intrahippocampal infusion of the NMDAR antagonist, AP5 (AP5 + HFS + LFT; *n* = 4). (3) LFT alone; *n* = 5, and (4) AP5 + LFT; *n* = 5. As shown in Figure 3A, LTP was blocked when HFS was applied in the presence of AP5. No changes in field potentials occurred in the LFT and AP5 + LFT treatment groups.

As a functional validation of LTP-specific gene expression in the dentate gyrus, qPCR analysis of *Arc* mRNA was performed. *Arc* is an immediate early gene of importance for protein synthesis-dependent synaptic plasticity and memory storage (Bramham et al., 2010). NMDAR-dependent *Arc* expression is tightly linked to LTP induction in the dentate gyrus (Messaoudi et al., 2007; Panja et al., 2009). *Arc* mRNA expression was elevated 34-fold in the HFS + LFT treated dentate gyrus relative to contralateral dentate gyrus and this increase was blocked by AP5-infusion and was absent in groups receiving LFT only (Figure 3B).

Eleven miRNAs from the high-throughput screen were chosen for qPCR validation. These selected miRNAs included the seven most strongly upregulated miRNAs (miR-330, miR-338, miR-223, miR-20a, miR-181a, miR-592, miR-212) in the Ago2 IP at 30 min, the only downregulated miRNA (miR-29b) in the Ago2 IP at 30 min, and the three most strongly upregulated miRNAs (miR-219, miR-384, let-7f) in the Ago2 IP at 120 min post-HFS (significant by *t*-test with Dunn–Bonferroni correction and 1-Way ANOVA with LSD test). For normalization of the qPCR data, miR-345-3p was used as the most stably expressed miRNA across samples. miR-181a levels were detected in the HFS-treated dentate gyrus but were below the detection limit in the contralateral (unstimulated) dentate gyrus. miR-181a was therefore excluded from quantitative analysis.

The results of the qPCR analysis of the 10 miRNAs examined are shown in Figures 3C–E. The Ct values obtained in treated dentate gyrus were normalized to control Ct values in the contralateral dentate gyrus for each rat. This was done separately for the input (Figure 3C) and Ago2 IP (Figure 3D) samples. Eight of the 10 miRNAs with altered expression in the Ago2 IP on the PCR card were similarly regulated by qPCR (Table A1). Overall, larger increases were obtained by TaqMan qPCR, which may reflect a wider dynamic range of Ct values obtained by cDNA amplification in the TaqMan analysis. Robust quantitative differences in miRNA expression were observed between input samples and Ago2 IP samples. Five miRNAs (miR-384, miR-29b, miR-219, miR-592, and miR-20a) exhibited 2 to 5-fold greater increases in expression in the Ago2 immunoprecipitate than in the input samples, relative to contralateral control values (Figures 3C,D). miR-330 and miR-223 expression was unchanged or slightly decreased in input samples at 30 min post-HFS but enhanced in the Ago2 IP. In contrast, miR-let7f and miR-338 exhibited 2-fold greater increases in the input sample compared to the Ago2 pellet. Finally, miR-212 was elevated in the input sample, but was significantly decreased in abundance in the Ago2 pellet.

Next we examined the role of NMDA receptor activation in regulation of miRNA expression. Local infusion of AP5 blocked LTP induction and prevented the increase in miRNA expression in input samples and Ago2 IP samples. Remarkably, all 10 miRNAs, including those that were not regulated or decreased in the HFS group, exhibited a significant decrease in expression when HFS was applied in the presence of AP5. No significant changes in miRNA expression were observed in Ago2 IP or input samples of the LFT or LFT + AP5 treatment groups. An analysis of the Ago2/input ratios revealed two distinct patterns of miRNA expression (Figure 3E). Seven miRNAs (miR-384, miR-29b, miR-219, miR-592, miR-20a, miR-330, and miR-223) showed enhanced, NMDAR-dependent association with Ago2. In contrast, 3 miRNAs (miR-let7f, miR-338, and miR-212) exhibited decreased expression in the Ago2 IP relative to input.

### Regulation of *Arc*-targeting miRNAs

**Figure 4:**
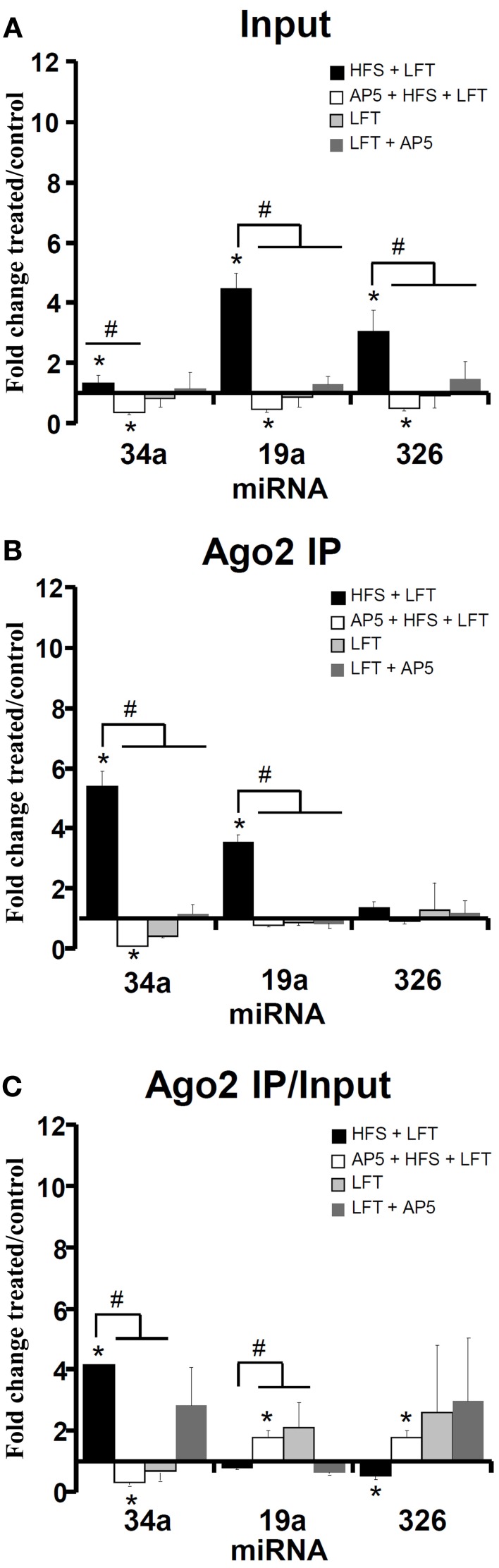
**Regulation of *Arc*-targeting miRNAs in LTP**. Quantitative PCR was used to examine the expression of a set of 3 *Arc*-associated miRNAs at 30 min. The same set of miRNAs were analyzed in the input sample **(A)** and Ago2 immunoprecipitate **(B)**. The qPCR data was normalized to the expression of miR-345-3p. **(A)** Fold change in *Arc*-associated miRNA expression in dentate gyrus lysates at 30 min post-HFS. Bar graph shows mean fold change (+s.e.m.) in treated dentate gyrus relative to control, contralateral dentate gyrus. HFS + LFT, *n* = 6; AP5 + HFS + LFT, *n* = 4; LFT alone, *n* = 5; AP5 + LFT, *n* = 5. Significant differences between the HFS group and other treatment groups are indicated (#; *p* < 0.05). Significant difference between the ispilateral (treated) and contralateral (control) dentate gyrus are indicated (^*^). **(B)** Fold change in *Arc*-associated miRNA expression in dentate gyrus Ago2 immunoprecipitates at 30 min post-HFS. Significant differences between the HFS group and other treatment groups are indicated (#; *p* < 0.05). Significant difference between the ispilateral (treated) and contralateral (control) dentate gyrus are indicated (^*^). **(C)** Relative fold change in *Arc*-associated miRNA expression in dentate gyrus Ago2 immunoprecipitates compared to dentate gyrus lysates (Ago2/input expression ratios) at 30 min post-HFS. Significant differences between the HFS group and other treatment groups are indicated (#; *p* < 0.05). Significant difference between the ispilateral (treated) and contralateral (control) dentate gyrus are indicated (^*^).

Recently, Wibrand et al. (2012) identified a set of miRNAs which bind to the *Arc* 3′ UTR and inhibit Arc protein expression in HEK293 cells and primary hippocampal neuronal cultures. Given the central role of Arc mRNA expression and translation in dentate gyrus LTP (Messaoudi et al., 2007; Panja et al., 2009), we were interested in determining the Ago2/IP expression pattern of Arc-targeting miRNAs. In input samples, all three miRNAs examined (miR-34a, miR-19a, miR-326) showed enhanced expression 30 min post-HFS and decreased expression below the contralateral control level when HFS was given in the presence of AP5 (Figure 4A). Analysis of the Ago2 IP and Ago2 IP/input expression ratios revealed enhanced NMDAR-dependent association of miR-34a with Ago2 make this (Figures 4B,C). In contrast, miR-19a showed no change and miR-326 had a significantly decreased Ago2/IP expression ratio following HFS. When AP5 was infused prior to HFS, the Ago2/IP expression ratio of miR-19a and miR-326 was increased, indicating NMDAR-dependent dissociation of these miRNA from Ago2 (Figure 4C).

### Stable expression of Argonaute 2 protein during LTP

**Figure 5:**
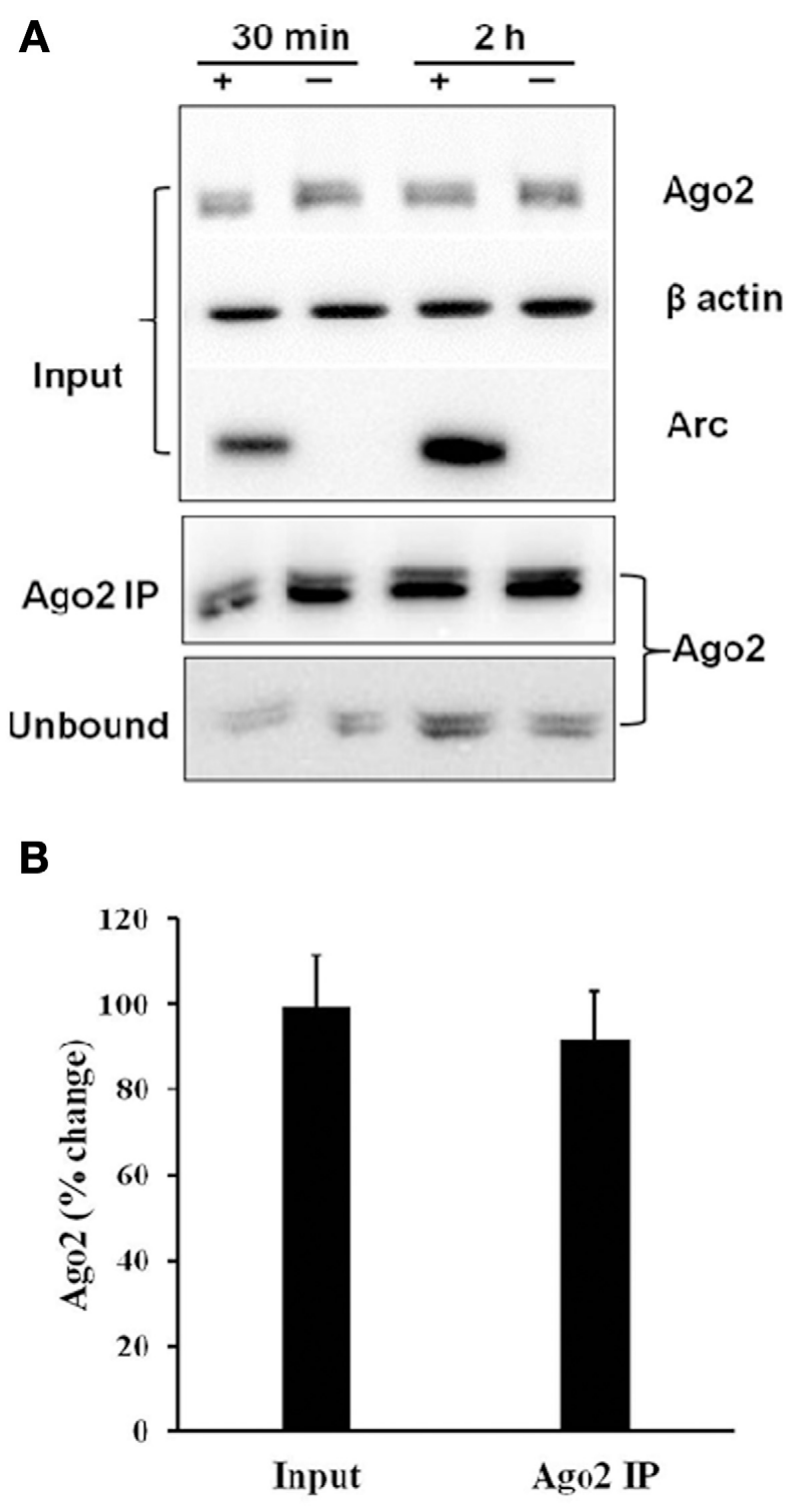
**Stable expression of Argonaute 2 protein during LTP. (A)** Immunoprecipitation of Ago2 30 and 120 min post-HFS. Representative immunoblot of Ago2 expression levels in input, Ago2 immunoprecipitate (Ago2 IP), and unbound fraction. **(B)** Bar graph shows mean % change (+s.e.m.) in Ago2 levels in input and Ago2-IP samples of treated dentate gyrus relative to the contralateral citation(untreated) dentate gyrus (*n* = 17).

Levels of Argonaute protein are known to influence the stability of miRNA (Winter and Diederichs, 2011). We therefore examined the expression of Argonaute protein as a potential mechanism for the differential regulation of total and Ago2-immunoprecipitated miRNAs. As shown in Figure 5, immunoblot analysis showed no change in Ago2 expression in input and Ago2-immunoprecipated samples at 30 and 120 min post-HFS (Figure 5).

### miRNA target gene prediction and pathway analysis

In order to better understand the potential downstream effects of the activity-dependent enhanced and depleted Ago2-associated miRNAs we integrated predictions from four of the most commonly used miRNA target prediction resources; DIANA (Maragkakis et al., 2011), miRanda (Griffiths-Jones et al., 2008), TargetScan (Friedman et al., 2009), and PicTar (Lall et al., 2006) using the RP method (Breitling et al., 2004). This allowed us to mitigate for the poor agreement normally found between different target gene prediction algorithms (Reyes-Herrera and Ficarra, 2012). Target gene list sizes for miRNAs with activity-dependent association with Ago2 for the 8 enhanced miRNAs were 97 (miR-20a), 156 (miR-219), 58 (miR-223), 114 (miR-29b), 30 (miR-330), 91 (miR-34a), 156 (miR-384), and 53 (miR-592) and for the 5 depleted miRNAs were 52 (let-7f), 55 (miR-338), 47 (miR-212), 255 (miR-19a), 32 (miR-326).

We next created three union target gene sets of 684 (enhanced), 418 (depleted), and 1019 (combined) predicted miRNA target genes to use as input for pathway enrichment analysis using the Bioconductor KEGGprofile package (Zhao, 2012). The combined list miRNA targets are strongly enriched in a number of key biological pathways relevant to activity-dependent synaptic plasticity (Table 1) including MAPK, mTOR, and Ras signaling pathways.

**Table 1:** **KEGG pathways targeted by the 13 activity-dependent miRNAs**.

**KEGG**	**Pathway**	***N***	***FC***	***p***
4360	Axon guidance	25	4.32	4.72E-11
4510	Focal adhesion	30	3.23	1.37E-09
4151	PI3K-Akt signaling pathway	35	2.28	5.02E-07
4320	Dorso-ventral axis formation	7	7.15	2.58E-06
4014	Ras signaling pathway	24	2.29	2.68E-05
4010	MAPK signaling pathway	25	2.15	5.46E-05
4150	mTOR signaling pathway	10	3.51	9.65E-05
4070	Phosphatidylinositol signaling system	11	3.01	2.27E-04
4350	TGF-beta signaling pathway	10	2.74	8.76E-04
4720	Long-term potentiation	8	2.77	2.06E-03
4144	Endocytosis	19	1.82	2.87E-03
4725	Cholinergic synapse	11	2.19	3.86E-03
4810	Regulation of actin cytoskeleton	17	1.74	6.99E-03
4722	Neurotrophin signaling pathway	11	2.01	7.52E-03
562	Inositol phosphate metabolism	7	2.35	8.92E-03
3018	RNA degradation	8	2.22	8.97E-03
4310	Wnt signaling pathway	12	1.90	9.12E-03
4120	Ubiquitin mediated proteolysis	11	1.82	1.58E-02
4340	Hedgehog signaling pathway	5	2.34	1.84E-02
3060	Protein export	3	2.81	2.00E-02
4728	Dopaminergic synapse	10	1.72	2.83E-02

*KEGG, KEGG accession number; N, number of target genes in pathway; FC, fold enrichment over expected*.

**Figure 6:**
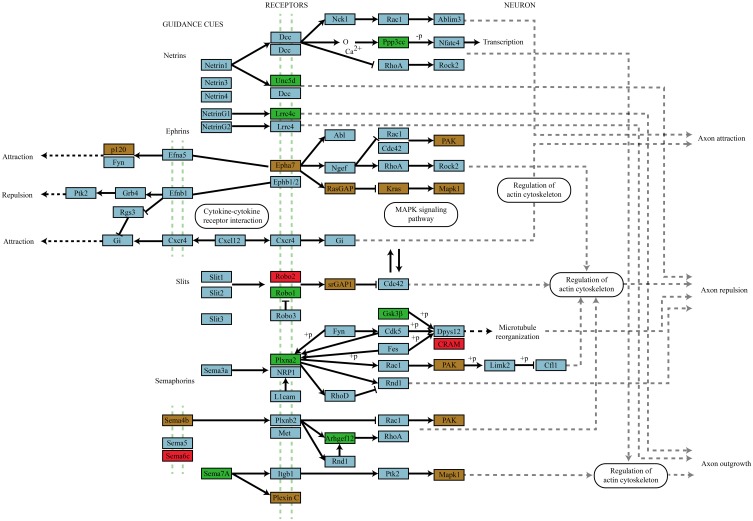
**Axon navigation pathway targeting by miRNAs that differentially associate with Ago2 in an activity-dependent manner**. Genes targeted by miRNAs that differentially associate with Ago2 in an activity-dependent manner are color coded by whether they are targeted by enhanced (green box), depleted (red box), or by both enhanced and depleted miRNAs (brown box). The involvement of all four guidance molecule families and their convergence on signal transduction systems classically associated with activity-dependent synaptic plasticity suggests an important role for these receptor mediated signaling pathways in plasticity.

Of particular note is the greater than 4-fold over-representation (*p* = 4.73 × 10^−11^) of genes traditionally involved in the mediation of axon guidance. Figure 6 shows a schematic of the axon navigation pathway annotated to show the targeting of enhanced and depleted miRNAs. The enhanced miRNA pool remarkably targets receptors of all four signaling families of the pathway; ephrins, netrins, semaphorins, and Slits as well as MAPK1 and GSK3β which have well-established roles in the regulation of activity-dependent synaptic plasticity. Both enhanced and depleted microRNAs appear to heavily target genes involved in ephrin signal transduction especially the cascade directly upstream of MAPK1. The only place where enhanced and depleted targets directly oppose each other is in differential targeting of the Robo1 and Robo2 receptors for Slit1/2.

Pathway enrichment analyses with the individual enhanced and depleted lists produce very similar pathway enrichment profiles to the combined lists (see Tables A2, A3).

## Discussion

microRNA levels are dictated by a multistep biogenesis pathway and probably multiple mechanisms for miRNA decay (Kai and Pasquinelli, 2010; Meister, 2013). Knowledge of miRNA expression during synaptic plasticity has so far relied on measurements in lysates from whole tissue or subcellular fractions. In the canonical biogenesis pathway, the guide strand of the mature miRNAs is bound by Ago to form the miRISC. Mature miRNA is considered to be present predominantly in tight complex with Argonaute. The present analysis of LTP in the dentate gyrus of anesthetized rats demonstrates differential regulation of mature miRNA expression in whole lysates and the Ago2 immunoprecipitated fraction. Both quantitatively and qualitatively, miRNA expression in tissue lysates does not accurately reflect changes in the miRNA content of the Ago2-RISC. The ratio of Ago2/total miRNA expression was regulated bidirectionally in a miRNA-specific manner and was largely dependent on NMDA receptor activation during LTP induction. The present results identify miRNA association with Ago2 as a potential control point in activity-dependent synaptic plasticity in the adult brain.

The high-throughput screen comparing HFS-treated and contralateral dentate gyrus indicated dynamic and differential regulation of total and Ago2-immunoprecipated miRNAs. A systemic validation using TaqMan qPCR demonstrated differential regulation at 30 min post-HFS. When comparing miRNA Ago2/input expression ratios, eight miRNAs (miR-384, miR-29b, miR-219, miR-592, miR-20a, miR-330 miR-223, and miR-34a) exhibited increases relative to the contralateral dentate gyrus, whereas five miRNAs (miR-let7f, miR-338, miR-212, miR-19a, and miR-326) showed decreases in this ratio. The results demonstrate activity-dependent, miRNA-specific regulation of miRNA abundance in the Ago2-RISC relative to total miRNA. The enhanced expression of miRNA and enhanced associated with Ago2 was specific to NMDA receptor-dependent LTP induction, as was the decrease in the Ago2/input ratio for miR-let7f and miR-326. These changes in miRNA expression were blocked when HFS was applied in the presence of NMDAR antagonist, AP5, and were absent when low-frequency test stimulation was applied alone or in combination with AP5 infusion. We therefore conclude that LTP induction is coupled to NMDAR-dependent regulation of miRNA expression and miRNA-specific interactions with Ago2. In addition, those miRNAs upregulated following HFS exhibited significant downregulation upon LTP block by AP5, which was not seen in the low-frequency test stimulation control groups. This indicates that HFS also acts through an unknown, NMDAR-independent mechanism to downregulate miRNA expression and its association with Ago2. Finally, this work does not exclude the possibility of small changes in miRNA expression in the contralateral dentate gyrus, although we have demonstrated LTP-specific regulation of miRNAs in the HFS-treated dentate gyrus.

Consistent with our previous work (Wibrand et al., 2010), expression of miR-212 and miR-132 was increased in dentate gyrus lysates at 120 min post-HFS. Wibrand and colleagues observed NMDAR-dependent decreases in mature miR-212 and miR-132 in the absence of NMDAR-dependent effects on precursor miRNA levels, suggesting that NMDAR signaling promotes decay of the mature miRNAs. Consistent with increased turnover, decreased expression was observed for ~50% of the miRNAs that were regulated in the input samples at 30 and 120 min post-HFS. However, the present work focused on qPCR validation of miRNAs that were upregulated in the Ago2 IP.

Park and Tang (2009) used miRNA arrays to determine the temporal expression profiles of 62 miRNAs in adult mouse hippocampal slices following induction of chemical LTP (C-LTP) and metabotropic glutamate receptor-dependent LTD (mGluR-LTD). The study demonstrated regulation of total (lysate) miRNA expression for the majority of miRNAs (50/61) by both C-LTP and mGluR-LTD. Most regulated miRNAs exhibited rapid upregulation at 15 min and declined thereafter but were still upregulated 2 h after LTP or LTD induction. In the present *in vivo* study, LTP was induced by patterned HFS of the medial perforant path input to the dentate gyrus. Rapid regulation at 30 min post-HFS was also observed in the present study, particularly in the Ago2 IP samples. However, the high-throughput screen shows delayed (2 h) bidirectional changes in miRNA expression in both Ago2 immunoprecipitates and lysates.

### Regulaton of Ago2-microRNA interactions

The differential regulation of Ago2 immunoprecipitated and total miRNA expression indicates the existence of a substantial non-Ago2-bound or “free” miRNA fraction. A key issue to be resolved is the nature of the non-Ago2-bound pool. This pool may consist of (1) mature miRNAs in the RISC loading complex ready to be loaded onto Ago, (2) miRNAs discharged from Ago following degradation of the target mRNA, (3) miRNAs weakly bound to Ago2 which dissociate during sample preparation, and (4) miRNAs bound to other members of the Argonaute family.

Co-expression of Argonaute family proteins (Ago1, 2, 3, and 4) occurs in many animal cell types including neurons. The four Ago proteins appear to play overlapping roles in mammals (Juvvuna et al., 2012; Meister, 2013). All mammalian Ago proteins contain PAZ, MID, and PIWI domains. The PAZ domain anchors the 3′-end of the miRNA, while the MID domain harbors a binding pocket for the 5′-end of the miRNA. The PIWI domain of Ago2, but not of other mammalian Ago proteins, has endonucleolytic activity capable of cleaving perfectly complementary sites. In mammals, however, miRNAs bind predominantly to partially complementary mRNA and Ago2-mediated “slicing” of target mRNA is uncommon. Rather, the mammalian Ago-RISC inhibits translation and promotes deadenylation-induced mRNA decay. Recent work suggests a sequential mechanism whereby rapid translation inhibition is followed by slow mRNA degradation (Béthune et al., 2012; Djuranovic et al., 2012).

Mammalian miRNAs are thought to be randomly sorted onto the four Argonaute proteins rather than targeted to specific Ago proteins (Dueck et al., 2012; Wang et al., 2012). If this is true for the brain, then analysis of Ago2 immunoprecipitated miRNAs can be considered representative of miRNA regulation as a whole. Wang et al. (2012) immunoprecipitated Ago1–3 from mouse keratinocytes and human melanoma cells and performed qPCR analysis to quantify the levels of miRNAs that interact with each Ago species. Ago2 interacted with a majority of miRNAs (60%), compared with Ago1 (30%) and Ago3 (<10%) in a proportion that matched the abundance of each Ago. However, processing of some miRNA precursors is mediated by Ago2 rather than Dicer. Dueck et al. (2012) demonstrated that ectopically expressed miR-451 in HEK293 cells is not only processed by Ago2 but also loaded exclusively onto Ago2-associated RISC. Once associated with Ago2, the mature miRNA is not exchanged with other Ago family proteins. In medium spiny neurons of the striatum, Ago2 (but not other Agos) contributes to the expression of some 25% of mature miRNAs (Schaefer et al., 2010). Therefore, it is possible that the Ago2 immunoprecipitated miRNAs identified in the present study are skewed toward Ago2 processing-dependent miRNAs. Immunoprecipitation of other Ago family proteins is needed to address the issue.

Recent work suggests that miRNA loading onto Ago is a regulated process. The minimal RISC loading complex consists of Dicer, the double-stranded RNA-binding protein, TRBP, heat-shock protein 90, and Ago. The current view is that miRNA loading onto Ago occurs in two-steps (Meister, 2013). The mature microRNA duplex is first bound by TRBP, which repositions the duplex on Dicer in an orientation that affords correct strand selection. The actual transfer of the miRNA duplex to Ago is mediated by heat shock protein 90 (HSP90), which binds Ago and keeps it in an open state capable of capturing the miRNA. Additional mechanisms likely exist for sequestering miRNA from Ago. For example, the RNA-binding protein, hnRNP E2, has been shown to reversibly sequester miR-328 away from Ago, Dicer, and other proteins of the RISC loading complex (Eiring et al., 2010).

A set of positive-charged arginine amino acids concentrated in the nucleotide-binding channel in Ago stabilizes the interaction of the protein with miRNA (Wang et al., 2010). However, molecular dynamic simulations and thermodynamic analysis indicate that conformational changes within the flexible PAZ domain could affect the recognition and release of miRNA (Wang et al., 2010). Recent evidence suggests that Ago2 is also extensively regulated by phosphorylation. A highly conserved tyrosine (Y529) located in the 5′-end-binding pocket of Ago2's MID domain can be phosphorylated (Rüdel et al., 2011). This phosphorylation inhibits small RNA binding to Ago2, suggesting that it may serve as a reversible molecular switch on miRNA binding. If Ago2 conformation is so regulated during LTP, this could provide a basis for the reversible modulation of Ago2:miRNA binding.

By stabilizing guide-stranded mature miRNA, Ago vastly extends the half-life of miRNA. Downregulation or ectopic expression of Ago results in decreased or enhanced miRNA levels, respectively (Winter and Diederichs, 2011). Hence, changes in Ago2 abundance would be expected to influence miRNA levels. However, modulation of Ago2 levels does not appear to be a contributing mechanism in LTP, as there was no change in the amount of total or immunoprecipitated Ago2 at 30 or 120 min after LTP induction.

Work in *C. elegans* suggests that target mRNA availability is a key factor in determining the release of miRNA from Argonaute and subsequent degradation of the miRNA (Chatterjee and Grosshans, 2009). miRNA binding to target stabilizes the miRNA:Ago interaction. miRNAs bound to abundant and stable mRNA will themselves be stable. Upon degradation of the target mRNA, miRNAs are released from Ago and degraded. Accordingly, changes in the mRNA expression profile during LTP are likely to influence the ratio of Ago2-bound to free miRNA in an miRNA-specific manner.

Circular RNAs (circRNAs) are a major class of regulatory RNAs which function to sponge endogenous miRNAs (Hansen et al., 2013; Memczak et al., 2013). CircRNAs are diverse (at least 2000 in human and mouse), abundantly expressed in brain, and contain numerous miRNA binding sites. miR-7 binding to a specific circRNA (csRS-7) results in robust derepression of miR-7 mRNA targets in neurons. Although miRNAs remain bound to Ago on circRNAs, circRNAs are resistant to miRISC-mediated destabilization. As a result, any movement or exchange of miRNAs between their binding sites on circRNA and mRNA is likely to shift the balance between free and Ago2-associated miRNA. Regulation of circRNAs in LTP is hypothetical at present, but could potentially contribute to rapid changes in the Ago2-miRNA pool.

A recent study in cortical synaptosomes suggests that axon terminals accumulate, store, and secrete miRNAs (Xu et al., 2013). Synaptosomes are biochemical fractions containing axon terminals that are pinched-off and released, often together with components of the postsynaptic membrane. The authors characterized miRNA profiles in synaptosomes using microarrays and qPCR. Endogenous Ago2-bound miRNAs were enriched in the synaptic vesicle fraction. Synthetic miR-125 was also taken up by the synaptosomes through a non-specific endocytic mechanism. Interestingly, the proportion of Ago2/total miRNAs in the synaptosome varied considerably among the 21 miRNAs, indicating that a miRNA-specific, non-Ago2-bound pool exists. Upon KCL-evoked depolarization, endogenous miRNAs are secreted from synaptosomes still attached to Ago2. It is therefore possible that secretion of Ago2 bound miRNAs, from postsynaptic or presynaptic compartments, alters the equilibrium between free and Ago2-bound miRNAs.

### miRNA target prediction and pathway analysis

Central to our understanding of miRNA function is the identification of their direct molecular targets. To date, no direct binding screens have taken place in experimental systems relevant to activity-dependent synaptic plasticity although techniques now exist to do so (Helwak et al., 2013). In the absence of such data we rely on miRNA target prediction algorithms that use structural, sequence and evolutionary based features of known miRNA-binding sites (Reyes-Herrera and Ficarra, 2012). Despite their widespread adoption, however, there is poor agreement between predictions made by different algorithms for the same miRNA. We adopted the RP approach to identify the most consistently predicted target genes from four of the most widely used miRNA target prediction algorithms. This produced mutually supportive target gene lists of on average 100 genes per miRNA that had lost 94.9% of predicted targets during the cross-comparison analysis by RP (Table A4).

Comparison of the biological pathways targeted by miRNAs with enhanced and depleted activity-dependent Ago2 association revealed 21 significantly enriched pathways (*p* ≤ 0.05). Among these were many previously reported to play roles in the regulation of activity-dependent synaptic plasticity in four broad categories including *remodeling and turnover pathways:* cell-cell adhesion (Gerrow and El-Husseini, 2006), actin cytoskeletal rearrangement (Bosch and Hayashi, 2012), endocytosis (Huganir and Nicoll, 2013; Jiang and Ehlers, 2013) proteolysis and protein export (Bingol and Sheng, 2011), *signal transduction pathways*: ERK/MAPK (English and Sweatt, 1997; Rosenblum et al., 2002; Ying et al., 2002; Kelleher et al., 2004), PI3K-Akt, mTOR (Hoeffer and Klann, 2010), Ras (Stornetta and Zhu, 2011), TGFβ, neurotrophin, and Wnt (Poon et al., 2013), *process pathways*: LTP, cholinergic and dopaminergic synapse activity and *developmental pathways*: axon guidance and dorso-ventral axis formation. This latter group show the highest and most significant enrichment in our miRNA targets and emerging evidence supports a central role for classical developmental pathways such as axon guidance in the regulation of synaptic plasticity (Wibrand et al., 2006; Knafo and Esteban, 2012). Indeed the intracellular signaling pathways through which netrins (Bayat et al., 2012; Horn et al., 2013), ephrins (Lim et al., 2008; Klein, 2009), Slits (Soderling et al., 2007), and semaphorins (Pasterkamp and Giger, 2009) operate are the same pathways found to be functional during activity-dependent synaptic plasticity.

### Closing comments

In sum, the present work provides evidence for bidirectional changes in miRNA expression compatible with regulated shuttling of miRNA to and from Ago2 in the adult dentate gyrus. As recent work has succeeded in cell-specific expression profiling of Ago2-associated miRNAs in brain (He et al., 2012), it will be important to elucidate the signaling pathways and cell biological mechanisms that dictate time-dependent interactions of miRNA with Argonautes. In addition, the bioinformatic predictions for biological processes modulated by Ago2-regulated miRNAs strongly point to regulation of mechanisms classically involved in axon guidance.

### Conflict of interest statement

The authors declare that the research was conducted in the absence of any commercial or financial relationships that could be construed as a potential conflict of interest.

## Appendix

**Table A1:** **Comparison of PCR card screening to Taqman qPCR validation results**.

**miRNA**	**miRNA levels in Ago2 IP following LTP induction**	
	**PCR card**	**qPCR**
384	↑	↑
29b	↓	↑
219	↑	↑
592	↑	↑
20a	↑	↑
let-7f	↑	↑
330	↑	↑
338	↑	↑
223	↑	↑
212	↑	↓
34a	−	↑
19a	−	↑
326	−	−

*Significantly increased expression (↑); significantly decreased expression (↓); no change (−)*.

**Table A2:** **Neural related pathways enriched in targets with decreased activity-dependent Ago2 association**.

**KEGG**	**Pathway name**	***N***	***FC***	***P***
4360	Axon guidance	19	4.71	1.93E-09
4510	Focal adhesion	24	3.70	3.23E-09
4151	PI3K-Akt signaling pathway	28	2.62	3.46E-07
4512	ECM-receptor interaction	11	4.07	1.23E-05
4150	mTOR signaling pathway	8	4.03	1.35E-04
4070	Phosphatidylinositol signaling system	9	3.54	1.91E-04
4010	MAPK signaling pathway	18	2.22	3.11E-04
4320	Dorso-ventral axis formation	4	5.86	4.66E-04
4014	Ras signaling pathway	16	2.19	7.16E-04
562	Inositol phosphate metabolism	7	3.37	9.74E-04
4728	Dopaminergic synapse	10	2.46	2.15E-03
3018	RNA degradation	7	2.78	3.29E-03
4350	TGF-beta signaling pathway	7	2.75	3.55E-03
4310	Wnt signaling pathway	9	2.04	1.14E-02
4720	Long-term potentiation	5	2.48	1.45E-02
5031	Amphetamine addiction	5	2.48	1.45E-02
4810	Regulation of actin cytoskeleton	12	1.76	1.57E-02
4330	Notch signaling pathway	4	2.53	1.98E-02
5034	Alcoholism	10	1.77	2.25E-02
4722	Neurotrophin signaling pathway	7	1.83	3.44E-02
4730	Long-term depression	4	2.08	4.15E-02

**Table A3:** **Neural related pathways enriched in targets with increased activity-dependent Ago2 association**.

**KEGG**	**Pathway name**	***N***	***FC***	***P***
4360	Axon guidance	13	6.01	2.13E-08
4320	Dorso-ventral axis formation	5	13.66	1.11E-06
4010	MAPK signaling pathway	14	3.22	1.77E-05
4540	Gap junction	7	4.73	9.98E-05
4014	Ras signaling pathway	12	3.07	1.01E-04
4725	Cholinergic synapse	7	3.72	5.10E-04
4720	Long-term potentiation	5	4.62	6.69E-04
4120	Ubiquitin mediated proteolysis	7	3.09	1.68E-03
4350	TGF-beta signaling pathway	5	3.66	2.23E-03
4722	Neurotrophin signaling pathway	6	2.93	3.96E-03
4530	Tight junction	6	2.61	7.31E-03
4730	Long-term depression	3	2.91	1.89E-02
4721	Synaptic vesicle cycle	3	2.86	1.99E-02
4510	Focal adhesion	7	2.01	2.00E-02
4150	mTOR signaling pathway	3	2.82	2.10E-02
4810	Regulation of actin cytoskeleton	7	1.91	2.60E-02
4310	Wnt signaling pathway	5	2.12	2.86E-02
4668	TNF signaling pathway	4	2.23	3.21E-02
4020	Calcium signaling pathway	6	1.91	3.40E-02
4340	Hedgehog signaling pathway	2	2.50	4.46E-02
4151	PI3K-Akt signaling pathway	9	1.57	4.82E-02
5034	Alcoholism	5	1.65	7.58E-02

**Table A4:** **Reduction in target gene list size resulting from rank product analysis**.

**miRNA**	**UTn**	**RPn**
let-7f	2195	52
miR-19a	1806	255
miR-20a	2244	97
miR-212	1365	47
miR-219	2205	156
miR-223	1274	58
miR-29b	2684	114
miR-326	1455	32
miR-330	2005	30
miR-338	2055	55
miR-34a	1588	91
miR-384	2064	156
miR-592	437	53
Total	23377	1196 (5.1%)

*UTn, Unique targets; RPn, Rank products*.
